# Effectiveness of 18F-FDG-PET/CT vs Bone Scintigraphy in Treatment Response Assessment of Bone Metastases in Breast Cancer

**DOI:** 10.1097/MD.0000000000003753

**Published:** 2016-05-27

**Authors:** Kusai M. Al-Muqbel, Rami J. Yaghan

**Affiliations:** From the Department of Radiology and Nuclear Medicine and the Department of Surgery, Faculty of Medicine, Jordan University of Science and Technology, Irbid, Jordan.

## Abstract

The aim of the study was to examine the effectiveness of fluorine-18 fluoro-2-deoxy-d-glucose positron emission tomography/computed tomography (18F-FDG PET/CT) versus bone scintigraphy (BS) in treatment response assessment of bone metastases in breast cancer.

The medical records of breast cancer patients with metastatic bone disease were reviewed retrospectively in our hospital from the period of January 2003 until April 2014. We included in our study patients evaluated by BS and/or 18F-FDG-PET/CT. Group 1 included patients who underwent pre- and post-treatment BS. Group 2 included patients who underwent pre- and post-treatment 18F-FDG-PET/CT scans. Group 3 included patients who underwent pretreatment BS and post-treatment both modalities. Functional and structural bone changes were monitored on pre- and post-treatment scans.

Group 1 included 71 patients, average age of 49.5 y (range 28–73 y). Post-treatment results were as follows: 34% stable disease, 43% progressed disease, 19% improved disease, 3% resolved disease, and 2% relapsed disease. Group 2 included 32 patients, average age 53.2 y (ranges between 37 and 78 y). Post-treatment results were as follows: 3% stable disease, 15% progressed disease, 15% improved disease, 53% resolved disease, and 14% relapsed disease. After treatment, the total symptomatic/imaging concordance rate was 51% in BS and 83% in 18F-FDG-PET/CT. Structurally, most patients with newly diagnosed metastatic bone disease had predominantly osteolytic lesions, which became mixed or osteoblastic after treatment as noted on CT images of responders. Group 3 included 8 patients, average age 48.9 y (ranges 32–64 y). Five patients had stable disease according to BS. 18F-FDG-PET/CT was concordant in 3/5 patients and discordant in 2/5 patients. Three patients had progressed disease on BS with concordant findings on 18F-FDG-PET/CT.

18F-FDG-PET/CT was found a powerful tool in treatment response assessment of bone metastases in breast cancer and consistent with clinical status of the patients as it reflects tumor activity. BS is insufficient for response assessment of bone metastases as it reflects osteoblastic reaction of the bone against metastatic disease which increases as the disease responds to treatment.

## INTRODUCTION

During the course of breast cancer, 30% to 85% of patients are diagnosed with bone metastases. The median survival duration after diagnosis of bone metastasis is 25.2 to 72 months. Serious skeletal-related events caused by bone metastasis—including fractures, spinal cord compression, and hypercalcemia—impair patient's quality of life. An accurate assessment of the disease condition and elimination of skeletal complications improve patient's quality of life.^1^ Accurate assessment of treatment response is vital to provide effective treatment as well as to avoid unnecessary treatment escalation. Early treatment response assessment of metastatic disease in breast cancer is important. Nonresponder could avoid unnecessary treatment toxicity and be switched to another potentially more effective treatment regimen if treatment response assessment is achieved early.^2–3^ Bone metastases are nonmeasurable by Response Evaluation Criteria In Solid Tumors (RECIST) criteria. The objective evaluation of their response to systemic treatment is difficult, and most clinicians rely primarily on the extent of symptomatic benefit to assess treatment response.^4^

Although bone scintigraphy (BS) is sensitive in detecting bone metastasis, changes in the appearance of bone lesions with effective treatment occur slowly, or even paradoxically, as exemplified in the phenomenon of bone scintigraphy “flare” making the evaluation of treatment difficult.^4^ Bone scintigraphy evaluates changes in bone structure rather than directly imaging the tumor, it can take as long as 6 months to reflect response to therapy.^1^

Fluorine-18 fluoro-2-deoxy-d-glucose positron emission tomography/computed tomography (18F-FDG PET/CT) is reported to be helpful in treatment response assessment and follow-up of metastatic bone disease in breast cancer patients.^4–6^ In metastatic setting, 18F- FDG-PET/CT contributes to management optimization by allowing termination of toxic therapies in nonresponders.^3^ However, the role of this modality in treatment response assessment of bone metastases in breast cancer is not sufficiently established.

The aim of the study was to examine the effectiveness of 18F-FDG-PET/CT and bone scintigraphy in treatment response assessment of bone metastases in breast cancer.

## PATIENTS AND METHODS

After obtaining hospital IRB approval, medical records of breast cancer patients with metastatic bone disease were reviewed retrospectively in our hospital (King Abdullah University Hospital/Irbid, Jordan) from the period of January 2003 until April 2014.

We included, in our study, breast cancer patients who developed metastatic bone disease and evaluated by bone scintigraphy and/or 18F-FDG-PET/CT. Patients were divided into 3 groups. Group 1 included patients who had undergone pretreatment baseline and post- treatment follow-up bone scintiscans. Group 2 included patients who had undergone pretreatment baseline and post-treatment follow-up 18F-FDG-PET/CT. Group 3 included patients who had undergone baseline bone scintiscan and post-treatment both modalities (bone scintigraphy and 18F-FDG-PET/CT).

Authors (1 nuclear physician and 1 surgical oncologist) had classified post-treatment bone scintiscans visually as resolved, improved, progressed, stable, or relapsed osteoblastic metastatic bone disease. *Resolved disease* is considered if the osteoblastic metastatic bone lesions visually disappeared completely. *Improved disease* is considered if there was a visual reduction in the intensity and/or in the number of osteoblastic metastatic bone lesions. *Progressed disease* is considered if there was a visual increase in the intensity and/or in the number of osteoblastic metastatic bone lesions. *Stable disease* is considered if there was no visual change in the intensity and/or in the number of osteoblastic metastatic bone lesions. *Relapsed disease* is considered if new osteoblastic metastatic bone lesions appeared after disease resolution.

Pre- and post-treatment 18F-FDG-PET/CT scans were evaluated visually and semi-quantitatively by 1 nuclear physician trained in PET/CT imaging with 5-year experience. Semiquantitative analyses were performed using the maximum standardized uptake value (SUVmax) calculated for the metastatic bony lesions according to the following formula:
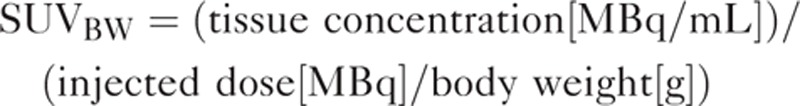


where BW = body weight, MBq = mega-Becquerel, mL = milliliter, g = gram.

The post-treatment change in SUVmax of the bony lesions was then calculated for each patient, allowing classification of patients according to European Organization for Research and Treatment of Cancer (EORTC) criteria^7^ into the following categories: *complete metabolic response* (complete resolution of bony tumor 18F-FDG uptake), *partial metabolic response* (at least 25% reduction in bony tumor 18F-FDG uptake), *stable metabolic disease* (<25% change in bony tumor 18F-FDG uptake with no change in lesions’ number and size), and *progressive metabolic disease* (>25% increase in bony tumor 18F-FDG uptake and/or new 18F-FDG-avid bony lesions). *Relapsed disease* was considered if new focal uptake appeared in bony skeleton after complete metabolic response.

As there is no acknowledged gold standard for assessing treatment response of bone metastases, we adopted a reference standard based on clinical assessment and laboratory results available for each patient for at least 6 months post-treatment.^8^

### Structural Types of Metastatic Bone Lesions Noted on PET/CT

The accompanying structural bone changes seen on CT part of PET/CT were classified as follows:

1. *Osteolytic:* bone destruction is noted on CT images.
2. *Mixed lesion:* bone destruction along with sclerotic component on CT images (mixed osteolytic and osteoblastic).
3. *Osteoblastic:* total sclerotic lesion on CT images.

Each individual patient had at least 2 scans including pretreatment baseline scan and 1 or more follow-up scans with potential different conclusions according to disease response to treatment and disease course. Hence, the same patient could be considered several times in our analysis.

First-line systemic chemotherapy consisted of 4 cycles of doxorubicin and cyclophosphamide followed by 4 cycles of docetaxel. Antiestrogen treatment consisted of tamoxifen in premenopausal patients and specific aromatize inhibitors in postmenopausal patients. HER-2-positive patients received trastusumab. Second-line and third-line chemotherapy were given according to current international guidelines.

### Imaging

#### PET/CT Scanning

All patients were asked to fast for at least 6 hours before 18F-FDG administration. Blood sugar was checked before 18F-FDG administration for all patients to confirm that it was <200 mg/dL. PET/CT acquisition was obtained using the PET/CT device (Discovery 600; GE Medical Systems, Milwaukee, WI). Whole-body scan was obtained from the base of the skull down to upper thighs. Unenhanced-CT scan was performed using a CT device with a 16-slice multi-detector helical scanner with the following standardized protocol settings: transverse 2.5-mm section thickness, 120 kVp, and 80 to 180 mA according to local body thickness. CT images were obtained for attenuation correction of PET images and for anatomical localization of F18-FDG distribution. PET scans were obtained 40 to 90 minutes after an intravenous administration of mean 260 Mbq (7 mCi) 18F-FDG. PET acquisition time was 2 to 3 minutes per bed position in the 2-dimensional mode. PET images were reconstructed with attenuation-weighted ordered-subset expectation maximization with and without attenuation correction.

#### Bone Scintigraphy

Whole-body bone scan was obtained after an intravenous injection of mean 740 MBq (20 mCi) Tc99m-diphosphonare (Tc99m-MDP). Whole-body planar images were obtained in anterior and posterior views 2 to 3 hours later and after voiding. Large-field-of-view gamma camera (Siemens E.CAM, IL) fitted with a low-energy and all-purpose parallel-hole collimator was used to obtain images. Photopeak was 140 KeV with a 15% pulse height analyzer (PHA) window.

### Statistical Analysis

The chi-squared test was used to compare post-treatment assessment types in group 1 and group 2 patients. *P*-value of <0.05 was considered significant. Analysis was performed by using the R software v 3.1.2.

## RESULTS

Group 1 included 71 patients, average age of 49.5 y (range 28–73 y), were assessed by 71 pretreatment bone scintiscans and134 post-treatment bone scintiscans. All patients had undergone 1 or more post-treatment bone scintiscans with an average interval of 6 months. Duration of follow-up was different from patient to patient as shown in Table 1. Number and percentage of post-treatment assessment types are shown in Table 2. Symptomatic/imaging concordance was also shown in Table 2. There is about 40% to 60% concordance rate between bone scintigraphy findings and symptomatic evaluation in most cases.

**TABLE 1:**
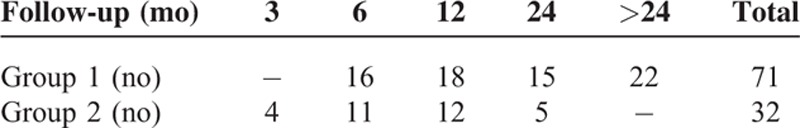
Duration of Follow-up in Group 1 and Group 2 Patients

**TABLE 2:**
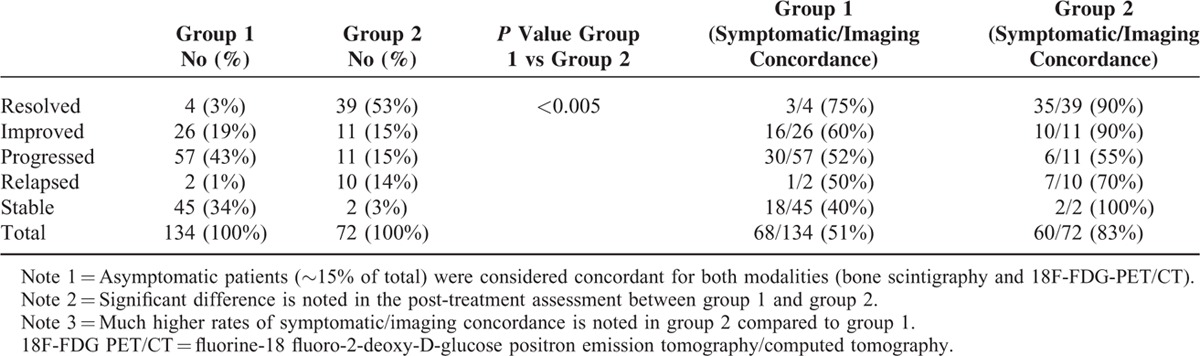
Post-Treatment Assessment in Group 1 and Group 2 Patients Sorted by Response Type. Symptomatic/Imaging Concordance Rate Was also Shown for Each Response Type for Both Groups

Group 2 included 32 patients, average age 53.2 y (ranges between 37 and 78 y). Half of them had new metastatic bone disease at time of staging 18F-18F-FDG-PET/CT, whereas the other half had history of treated bone metastasis at time of first follow-up 18F-FDG-PET/CT. The patients were assessed by 32 pretreatment 18F-FDG-PET/CT scans and 73 post-treatment 18F-FDG-PET/CT scans. All of them had undergone 1 or more post-treatment 18F-PET/CT scan (s) with average interval of 3 to 6 months. Duration of follow-up was different from patient to patient as shown in Table 1. Pretreatment SUVmax for metastatic bony lesions ranged between 3 and 22 (average SUVmax was 7.6). Number and percentage of post-treatment assessment types are shown in Table 2, which is statistically different than that for group1 (*P* value <0.005). The symptomatic/imaging concordance rate was also shown in Table 2. There is ∼90% concordance rate between 18F-FDG-PET/CT findings and symptomatic evaluation in those patients who had resolved/improved disease. On the other hand, the concordance rate was 55% to 70% in those patients with progressed/relapsed disease.

**FIGURE 1:**
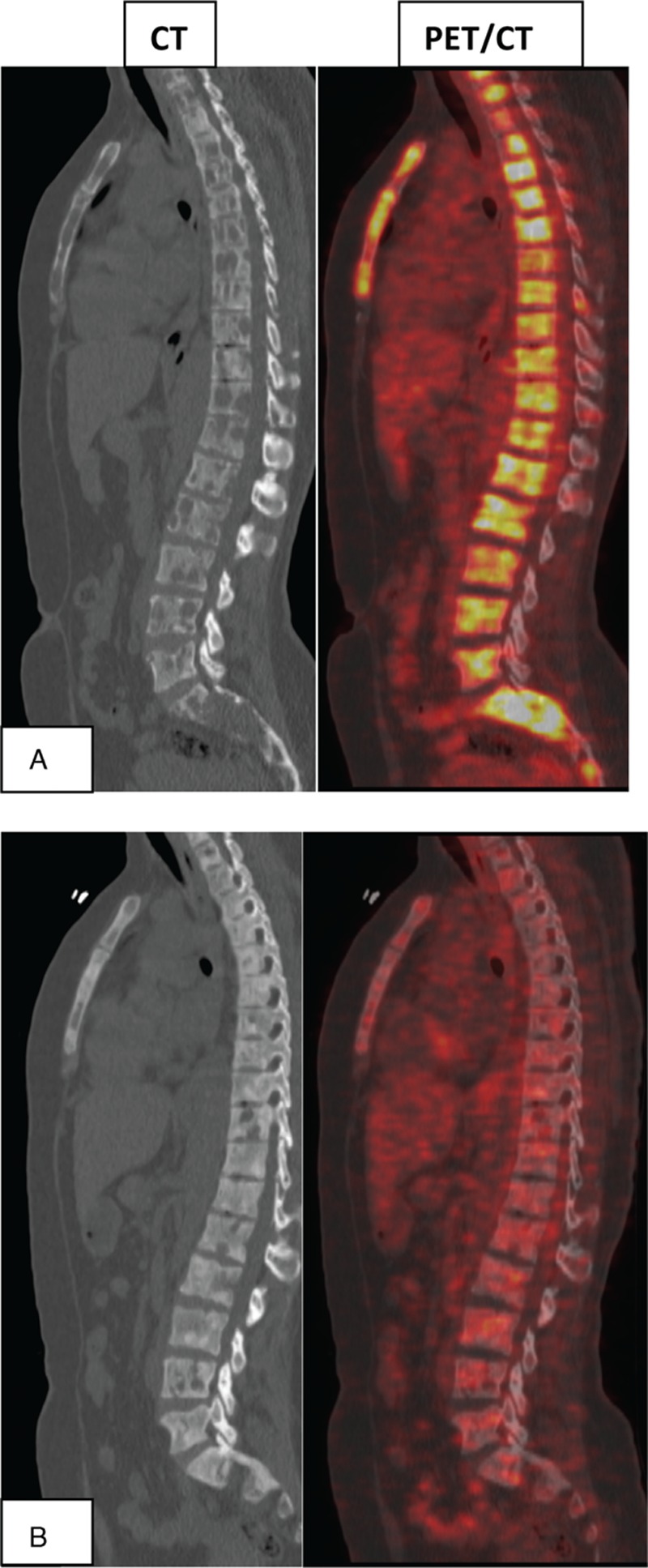
Multiple 18F-FDG-positive osteolytic metastatic lesions on baseline PET/CT (A) followed by complete response to treatment evidenced by total disappearance of 18F-FDG focal activity on follow-up PET/CT with ossification of osteolytic lesions that became osteoblastic lesions on CT images (B). 18F-FDG = fluorine-18 fluoro-2-deoxy-D-glucose, PET/CT = positron emission tomography/computed tomography.

Structurally, most patients with newly diagnosed metastatic bone disease had predominantly osteolytic lesions that became mixed or osteoblastic after treatment as noted on CT part of the examination (Figure 1). Most patients who did not respond to treatment clinically and metabolically persisted to have predominance of osteolytic bony lesions with no significant ossification as noted on CT images.

**FIGURE 2:**
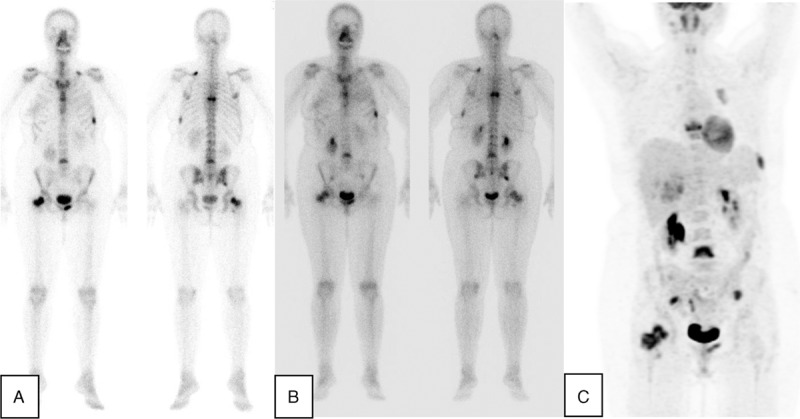
Baseline bone scintiscan with osteoblastic metastatic bone lesions in breast cancer patient (A), stable post-treatment bone scintiscan (B) and 18F-FDG-avid same metastatic bone lesions in post-treatment 18F-FDG-PET scan (C). Note: post-treatment scans were performed within 2-week interval window. 18F-FDG PET/CT = fluorine-18 fluoro-2-deoxy-D-glucose positron emission tomography/computed tomography.

Group 3 included 8 patients, average age 48.9 y (ranges 32–64 y). First, comparison was made between pretreatment and post-treatment bone scintiscans. Second, correlation was made between post-treatment bone scintiscans and post-treatment 18F-FDG-PET/CT scans provided that both studies were performed within 30 days window. Five patients had stable disease according to bone scintigraphy. At the same time, 18F-FDG-PET/CT was concordant in 3/5 patients and discordant in 2/5 patients (Figure 2). Three patients had progressed disease on bone scintigraphy with concordant findings on 18F-FDG-PET/CT.

## DISCUSSION

18F-FDG-PET/CT and bone scintigraphy exploit different mechanisms to detect tumor involvement in bone skeleton. Bone scintigraphy relies on an osteoblastic bone response to tumor, whereas 18F-FDG-PET/CT measures glucose uptake into the tumor itself. Bone scintigraphy indirectly reflects the biological events that take place with the onset of bone metastases, and thus can significantly underestimate or overestimate the actual response. Therefore, 18F-FDG-PET/CT is more likely to detect metastatic disease activity than bone scintigraphy.^9^

Our data showed that bone scintigraphy is not accurate in monitoring metastatic bone disease response to treatment in breast cancer. Bone scintigraphy persistently demonstrates osteoblastic metastatic activity in nearly all breast cancer patients with metastatic bone disease whether the disease is controlled or uncontrolled. The symptomatic/imaging concordance rate in most patients imaged by this modality ranged between 40% and 60%. The most frequent findings on follow-up bone scintigraphy in responders were “improved disease” and “stable disease,” whereas “progressed disease” and less frequently “stable disease” were the most frequent findings in nonresponders. In 1 study, metastatic bone lesions response to treatment was monitored by bone scintigraphy. The results were similar to ours as 92% of their patients continued to have abnormal bone scintiscans (8% complete response, 41% partial response, 31% stable disease, and 20% disease progression).^10^

On the other hand, our data showed that 18F-FDG-PET/CT was accurate in monitoring treatment response in metastatic bone disease in breast cancer patients. There was excellent symptomatic/imaging concordance rate in patients who had “resolved/improved disease.” In addition, 18F-FDG-PET/CT was able to detect “disease relapse/progression” early on even before symptoms’ appearance, which justifies the intermediate symptomatic/imaging concordance (55–70%) in these patients. This is because persistent focal metabolic activity means uncontrolled disease, whereas resolution of metabolic activity means disease control regardless of bone scintigraphic appearance or structural bone appearance on CT part of PET/CT.

Structurally, the accompanying CT part of PET/CT added more valuable information supporting the metabolic changes seen on PET part. Most patients initially had osteolytic lesions that ossified partially or completely after treatment converting into mixed lesions and/or osteoblastic lesions. Hence, ossification of osteolytic metastatic lesion is found to be a healing sign. Progressed or relapsed disease was remarkable for persistent and/or new osteolytic lesions on CT part of the exam.

Several reports were found in the literature assessing treatment response of metastatic bone disease in breast cancer by sequential 18F-FDG-PET/CT. Specht et al found 18F-FDG-PET/CT predictive of time-to-progression as patients with no change in SUV was twice as likely to progress compared to a patient with a 42% median decrease in SUV.^4^ Constantinidou et al found a strong relationship between response and decrease in uptake even at an early stage of therapy.^3^ Katayama et al found the change in the tumor marker levels was substantially correlated with the PET findings and moderately correlated with the CT findings.^11^

Post-treatment sclerosis of osteolytic lesions noted on CT part of 18F-FDG-PET/CT is responsible for persistent focal activity on bone scintiscan even in responders. Persistent focal uptake on bone scintiscan is misleading as both responders and nonresponders share similar findings. In keeping with that, several studies showed that an increase in the sclerotic component of the lytic lesions as assessed on the accompanying CT image contributed to the accuracy of a favorable response.^6,11, –12^

Regarding single-photon emission computed tomography (SPECT) role, we did not employ SPECT in bone scintigraphy acquisition in our study, which might theoretically create difficulty in comparing planar bone scintigraphy with PET. PET is a method that routinely includes tomography, whereas bone scintigraphy is not. Rather, SPECT in routine work is an elective option for local equivocal abnormality on the planar image. The practical value of bone scintigraphy is being a whole-body planar screening image, and it is difficult to do whole-body SPECT, which is time and labor consuming. Moreover, SPECT may play a role in lesion detection during diagnosis work-up, whereas in treatment response assessment, it is not helpful as we evaluated lesions that already visible on planar images.

From above discussion, our report confirms the effectiveness of 18F-FDG-PET/CT and ineffectiveness of bone scintigraphy in assessment of treatment response of bone metastases in breast cancer as reported by other researchers. However, each previously published report had studied 1 modality and up to the best of our knowledge we could not find in the literature a report comparing both modalities as we did.

## CONCLUSION

18F-FDG-PET/CT was found a powerful tool in treatment response assessment of metastatic bone disease in breast cancer and consistent with clinical status of the patients. 18F-FDG-PET/CT seems to be accurate in directing treatment of metastatic bone disease as it reflects tumor activity, which is structurally difficult to be assessed by CT scan alone or by bone scintigraphy. The latter reflects bone reaction against metastatic tumor which increases as the disease responds to treatment.
